# Electro-Mechanical Alterations in Atrial Fibrillation: Structural, Electrical, and Functional Correlates

**DOI:** 10.3390/jcdd10040149

**Published:** 2023-03-31

**Authors:** Iraklis Pozios, Apostolos Ilias Vouliotis, Polychronis Dilaveris, Constantinos Tsioufis

**Affiliations:** 1st Department of Cardiology, Hippokration Hospital, School of Medicine, National & Kapodistrian University of Athens, 11527 Athens, Greece

**Keywords:** atrial conduction, structural remodeling, fibrosis, Connexins, interatrial block, P wave parameters, voltage mapping, atrial dilatation, total atrial conduction time

## Abstract

Atrial fibrillation is the most common arrhythmia encountered in clinical practice affecting both patients’ survival and well-being. Apart from aging, many cardiovascular risk factors may cause structural remodeling of the atrial myocardium leading to atrial fibrillation development. Structural remodelling refers to the development of atrial fibrosis, as well as to alterations in atrial size and cellular ultrastructure. The latter includes myolysis, the development of glycogen accumulation, altered Connexin expression, subcellular changes, and sinus rhythm alterations. The structural remodeling of the atrial myocardium is commonly associated with the presence of interatrial block. On the other hand, prolongation of the interatrial conduction time is encountered when atrial pressure is acutely increased. Electrical correlates of conduction disturbances include alterations in P wave parameters, such as partial or advanced interatrial block, alterations in P wave axis, voltage, area, morphology, or abnormal electrophysiological characteristics, such as alterations in bipolar or unipolar voltage mapping, electrogram fractionation, endo-epicardial asynchrony of the atrial wall, or slower cardiac conduction velocity. Functional correlates of conduction disturbances may incorporate alterations in left atrial diameter, volume, or strain. Echocardiography or cardiac magnetic resonance imaging (MRI) is commonly used to assess these parameters. Finally, the echocardiography-derived total atrial conduction time (PA-TDI duration) may reflect both atrial electrical and structural alterations.

## 1. Introduction

**Figure 1:**
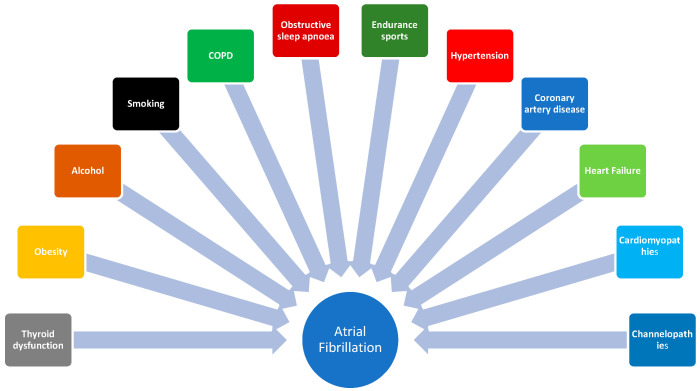
Common causes of atrial fibrillation. COPD: chronic obstructive pulmonary disease.

Atrial fibrillation (AF) is the most common arrhythmia encountered in clinical practice affecting both patients’ survival and well-being. [1]. The pathophysiological basis of AF includes four types of alterations, namely: (1) ion channel dysfunction, mainly a decreased plateau outward K+ current or decreased inward L-type Ca^2+^ accelerating repolarization (2) Ca^2+^-handling abnormalities, (3) autonomic neural dysregulation and, (4) structural remodeling [2]. All these alterations may promote ectopic firing and reentrant mechanisms [2,3,4,5]. The evolution of paroxysmal AF to the more permanent forms of arrhythmia lead to AF electrical remodeling, which is altered physiological properties and functions of the atrial tissue, such as the density and kinetics of ion channels [6]. On the other hand, the term structural remodeling refers to alterations in atrial tissue properties induced by aging and a series of cardiovascular risk factors [7]. Lifestyle factors such as alcohol consumption, smoking, obesity, physical inactivity, or participation in endurance sports, as well as common cardiovascular diseases such as hypertension, coronary artery disease, heart failure, cardiomyopathies, or channelopathies, play a significant role in the provocation of AF (Figure 1). Treatment of concomitant cardiovascular or other diseases and prevention of cardiovascular risk factors constitute an important pillar in the ABC pathway of AF therapy [8,9,10,11,12,13,14].

## 2. Structural Correlates of Conduction Disturbances

Structural remodelling refers to the development of atrial fibrosis, as well as to alterations in atrial size and cellular ultrastructure. The latter includes myolysis, the development of glycogen accumulation, altered Connexin expression, subcellular changes, and sinus rhythm (SR) alterations [2].

### 2.1. Atrial Fibrosis as a Hallmark Alteration in AF

Atrial fibrosis is the most important feature of structural remodeling in AF, although many inconsistencies in the association between fibrosis and AF are encountered [15]. However, in this context, it is assumed that reactive or interstitial fibrosis leads to the development of collagen septa both between muscle bundles (perimysial fibrosis) and myocytes (endomysial fibrosis). Therefore, interstitial fibrosis affects more severely atrial conduction as compared to replacement fibrosis [15,16].

**Figure 2:**
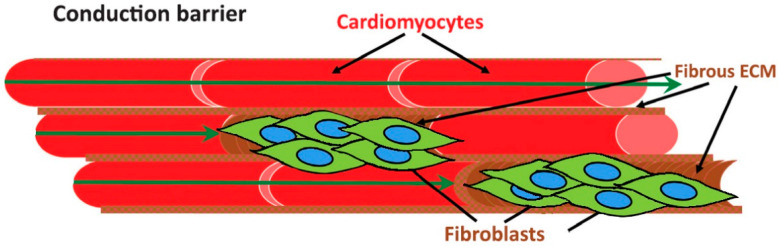
The role of fibroblast–cardiomyocyte interaction for the development of conduction barriers in the atrial myocardium.

Atrial fibrosis may promote arrhythmogenesis in many ways. First, it separates atrial fibers in the longitudinal direction and creates significant conduction barriers. Such conduction barriers are responsible for significant conduction delays or blocks that induce re-entry [2]. Second, atrial fibrosis in association with fibroblast proliferation and the development of fibroblast–cardiomyocyte interactions may result in conduction delays, abnormalities in action potential duration, and the appearance of delayed afterdepolarizations [17] (Figure 2). Quantitative imaging of atrial fibrosis by use of magnetic resonance imaging (MRI) tools could be combined with mathematical modeling to clarify the mechanisms by which atrial fibrosis leads to clinical AF [18,19]. An expert consensus document previously defined atrial cardiomyopathy as the presence of any complex structural, architectural, contractile, or electrophysiological changes affecting the atria with a significant impact on atrial function and arrhythmogenesis [20]. As a first attempt to characterize atrial pathologies into discrete groups, four different classes were defined: (I) primarily cardiomyocyte changes; (II) primarily fibrotic changes; (III) combined cardiomyocyte-fibrotic changes; (IV) primarily non-collagen infiltration [20]. Because disease-related atrial histopathology is often poorly characterized and commonly nonspecific, its classification may be challenging.

### 2.2. Altered Connexin Expression

Connexins are specialized hemichannel subunit proteins that maintain the low-resistance intercellular coupling and, therefore, the myocardial electric continuity. Connexin (Cx)43 and Cx40 are important for the atrial gap junctions [21]. Cx43 is usually localized at the end-to-end connections between atrial myocytes. In AF, Cx43 is translocated to the lateral myocardial membranes leading to slower conduction velocity or even to conduction block [22]. Gene therapy targeting atrial Cx43 expression showed very promising results in restoring the already depressed atrial Cx43 protein levels [23].

### 2.3. The Role of Interatrial Conduction 

The role of inter-atrial conduction in the development and management of AF is incompletely clarified [24,25]. Scarce anatomical studies in man or electro-anatomical mapping data in normal hearts [26] have proposed the presence of two major inter-atrial activation routes: the anterior inter-atrial band described by Bachmann [27], located in the anterior wall of the left atrium (LA) and connecting the right and left atrial appendages; the posterior interatrial route. However, variable connections between the coronary sinus (CS) and LA have been consistently shown by Chauvin et al. [28].

The associations between the presence of interatrial block (IAB) and structural remodeling of the atrial myocardium in the tissue, cellular, or subcellular levels are consistently shown [29]. IAB commonly appears in the fibrotic atria. Advanced IAB has been linked to extensive biatrial fibrosis detected on MRI that involves the Bachmann’s region [30]. During SR or pacing, patients with persistent AF may show slower conduction velocity compared with patients with paroxysmal AF in the LA and Bachmann’s bundle (BB) area [31,32]. In contrast, no differences in the conduction velocity between the SR and AF groups in the pulmonary vein area (PVA) were encountered [33]. Moreover, persistent AF is related to a higher number of conduction disorders at BB, prolonged P wave duration, and lack of spontaneous termination [34]. 

Aging is associated with conduction slowing and frequent conduction blocks, mainly at the BB and right atrium (RA) [29]. Low voltage areas are more evident at BB, RA, and PVA in older patients, as well as a decrease in the wavefront propagation velocity [35]. Fractionated electrograms [36,37], electrical uncoupling of side-to-side connections [38], and altered Connexin expression [39] were demonstrated in older patients.

### 2.4. The Effect of Stretch on Atrial Conduction

Recent studies have postulated the depressive effect of stretch on atrial conduction, focusing on the prolongation of interatrial conduction time when atrial pressure is acutely elevated [40]. Stretch may exert its effects on the electrical properties of the myocardial membrane, as well as the conduction velocity of the atrial tissue. Stretch slows conduction mainly through a reduction of cell excitability [41,42] and/or an increase in membrane capacitance [43]. Stretch has been shown to induce after-depolarizations and triggered arrhythmias, enhanced firing of the pulmonary veins [44,45], decrease in the refractory period, and increased dispersion of refractoriness [40].

## 3. Electrical Correlates of Conduction Disturbances

### 3.1. Alterations in P Wave Parameters

P wave parameters can undergo changes under various normal physiological conditions but more frequently when atrial pathology is present. These alterations can be easily detected on a standard 12-lead electrocardiogram (ECG) and can be measured manually or automatically (Table 1).

Advanced IAB has been associated with the development of AF or atrial flutter. Other P wave parameters, such as the P wave axis, P wave voltage, P wave area, and P terminal force in V1, have also been considered risk markers of AF [46].

#### 3.1.1. P Wave Duration

A P wave duration ≥ 120 ms is considered abnormal and is a criterion for partial IAB, that is a conduction delay between RA and LA through the BB. In the case of advanced IAB, the LA is retrogradely activated via muscular bundles located close to the atrioventricular junction. In advanced IAB, increased P wave duration is accompanied by P wave morphology changes in leads II, III, and aVF (i.e., biphasic). P wave duration independently predicts AF recurrence after radiofrequency (RF) ablation. The risk of AF recurrence increases substantially with a P wave duration > 150 ms [47].

#### 3.1.2. P Wave Axis

P wave axis values between 0° and 75° are considered normal. The abnormal P wave axis increased the risk of AF by 17% [48].

#### 3.1.3. P Wave Voltage

P wave voltage ≤ 0.1 mV in lead I is considered abnormal. Abnormally decreased P wave amplitude in lead I is associated with post-ablation AF recurrence [49].

#### 3.1.4. P Wave Area

Abnormal P wave area is defined as ≥4 ms×mV and has been considered a marker of left atrial enlargement (LAE) [50]. P wave area is calculated in lead II. The following formula is used: P wave area = ½P wave duration × P wave voltage.

#### 3.1.5. P Wave Terminal Force in Lead V1

The posterior displacement of the LA in LAE is manifested on the ECG with a longer P wave duration and a more pronounced negative component of the P wave in V1 [47].

#### 3.1.6. P Wave Dispersion

P wave dispersion is defined as the difference between the maximum and the minimum P wave duration in any of the 12 ECG leads. Higher values of P wave dispersion have been associated with a higher incidence of “lone” AF and the appearance of post-cardioversion AF recurrences [51].

### 3.2. Abnormal Electrophysiological Characteristics

Premature atrial contractions (PACs) may trigger AF initiation. PACs commonly originate from the pulmonary veins (95%) and scarcely from the superior vena cava and the coronary sinus, especially when associated with the persistence of the left superior vena cava (5%). Although pulmonary vein isolation (PVI) is a satisfactory ablation strategy, AF recurrences frequently occur after a successful PVI. The main reason for post-ablation AF recurrences is PV reconnection [52]. 

Progression from paroxysmal to more persistent forms of AF indicates the evolution from a trigger-mediated to an electro-pathology-mediated initiation and maintenance of the arrhythmia. Several electrophysiological characteristics have been proposed as indicators of AF-related electro-pathology at both atria and the BB.

#### 3.2.1. Bipolar Voltage Mapping

**Figure 3:**
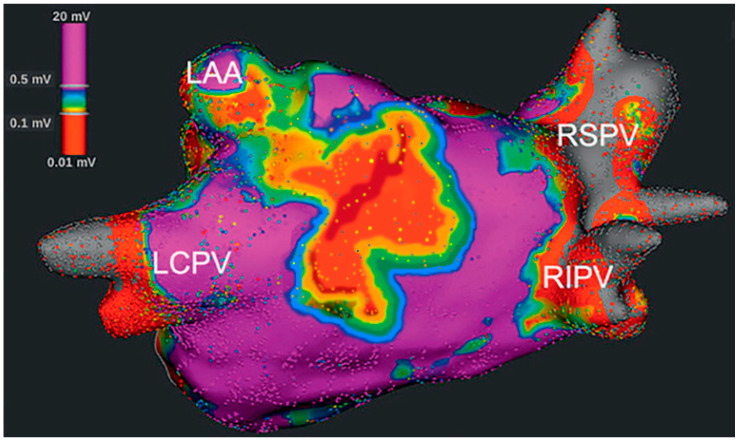
Low voltage zones in the LA are depicted during high-density bipolar voltage mapping. Bipolar voltage areas ≤ 0.1 mV are red, and those with voltage > 0.5 mV are purple, with interpolation of color for all the intermediate amplitudes.

The presence of low voltage areas (≤0.5 mV) in LA during high-density bipolar voltage mapping indicates the presence of fibrosis, is considered responsible for the initiation and maintenance of AF [32] (Figure 3).

Modern ablation strategies may combine PVI with LA substrate modification targeting low-voltage zones in the LA [53]. Rolf et al. used SR voltage mapping to guide AF substrate modification, mainly in patients with persistent AF [53]. The efficacy and the long-term outcome of LA substrate modification, although beneficial in some patients, remain still controversial [54].

#### 3.2.2. Unipolar Voltage Mapping

Unipolar electrograms (EGMs) provide additional information on wavefront progression. Unipolar voltages are higher in the LA compared to the RA in patients with persistent AF during CS pacing [55]. Unipolar voltage is lower in areas of slowed conduction or conduction block and areas containing fractionated potentials [56]. Moreover, low-voltage potentials have been recorded at the BB in the paroxysmal AF group compared to the non-AF group in patients with mitral valve disease.

#### 3.2.3. Electrogram Fractionation

Fractionated EGMs are associated with slow conduction. However, they may appear during SR in areas characterized by normal voltages and cardiac conduction velocity [57].

#### 3.2.4. Endo-Epicardial Asynchrony of the Atrial Wall

The presence of asynchronous activation of the atrial endocardial and epicardial layers may lead to focally propagating fibrillation waves [58]. Unipolar EGMs may detect fractionation-based endo-epicardial asynchrony more efficiently than bipolar ones [59].

#### 3.2.5. Cardiac Conduction Velocity

Isochronal maps in isopotential lines are drawn over a fixed time interval and are used to estimate conduction velocity. Conduction velocity has been shown to be slower in patients with AF [60]. Moreover, in patients with persistent AF, conduction velocity in RA was lower compared to LA [55]. Moreover, Zheng et al. showed that patients with AF, compared with those with atrioventricular nodal reciprocating tachycardia, are associated with conduction delay in both atria. This delay was found to be more evident in LA than in RA [61]. Prolonged local bipolar endocardial EGMs have been found in patients with lone paroxysmal AF and normal atrial volumes and voltages and were associated with interatrial conduction time (IACT) [62]. 

## 4. Functional Correlates of Conduction Disturbances

Atrial remodelling is assessed using echocardiography or cardiac MRI to measure LA size and function. Both are well-established predictors of AF-related outcomes.

### 4.1. Left Atrial Size and Strain

Atrial dilatation’s relationship to the incidence of AF was first described in mitral valve disease patients [63]. Twenty years later, another study showed that a LA diameter above 45 mm would predict AF recurrence over the 6 months following cardioversion in patients with valve disease or septal hypertrophy [64]. Left atrial enlargement was established as an independent risk factor of AF in two large prospective trials [65,66]. Animal models of acute atrial dilatation in both isolated hearts and in situ showed a decrease in the atrial refractory period [67,68]. On the other hand, no change [69] or even prolongation of atrial refractoriness was reported elsewhere [70,71]. All of them found an increment in the inducibility and persistence of AF, though. 

A change in atrial structure and function is the background of atrial remodelling, which results in left atrial enlargement [72,73]. On the other hand, conditions such as hypertension, structural, and valve heart disease may also induce similar changes in atrial anatomy or function because of an increase in LA pressure and/or volume. Atrial remodelling provides the substrate for AF episodes to be triggered, which results in a vicious circle of further atrial remodelling as a consequence [74,75]. 

It is well established that LA diameter is an independent predictor of AF recurrence post-PVI [76]. Although used widely, the LA anterior–posterior diameter is not the most accurate index of its “true” size [77]. Left atrial enlargement occurs in an asymmetric way and is mainly oriented in the superior–inferior and medial–lateral directions [78]. In a study assessing LA volume by computed tomography, it was found to be related to the outcome of RF ablation, whereas the echo-derived LA anterior–posterior diameter was not [79]. Cardiac MRI is a more accurate modality compared to echo in order to assess LA volume and is also being used to assess the arrhythmic substrate in regard to catheter ablation [80]. Patients with AF have larger LA volumes, as assessed by MRI, compared to those with SR [81,82]. On the contrary, those with “lone AF” have similar LA volumes to healthy individuals [83]. Additionally, persistent versus paroxysmal AF is related to higher LA volumes [84].

Atrial contractile dysfunction, as assessed by echocardiography, is correlated with the duration of AF. After a 2-week period of AF and restoration of SR, 24 h was the time for the recovery of atrial contractile function. On the other hand, it took more than 1 month for recovery, while AF lasted more than 6 weeks [85,86]. A new atrial thrombus can be formed after cardioversion or even several days or weeks later [87]. Thus, the time needed for a full recovery of atrial contraction after the restoration of SR may affect the occurrence of thromboembolic events [88]. It is not clear what mechanisms are involved in post-fibrillatory contractile dysfunction. Initially, it was thought that electrical cardioversion caused “atrial stunning” [87], but soon it was revealed that, even after the pharmacological and spontaneous restoration of SR, the atrial contractile function was depressed [89,90].

In another animal model of AF [91], changes in atrial contractility, as expressed by a reduction of the atrial work index and a shift in atrial electrical properties with a shortening of the atrial effective refractory period, followed the exact same time course. It is known that the main cellular mechanism of electrical remodelling is the reduction of the L-type Ca^2+^ inward current (I_CaL_) [92]. As a result, it is thought that this reduction of I_CaL_ induced the atrial contractile dysfunction noted during the first 5 days of AF. Interestingly, after the restoration of SR, atrial contractility recovered completely within 3 days. An increase in compliance and the diameter of the atrium was noted early, during the first days of AF, following the same time course as the loss of contractility, and were fully reversible within two days of SR. Loss of atrial contractility is one of the mechanisms of atrial dilatation during prolonged AF, while the stretch of the atrial wall might lead to the elongation of the collagen fibers. Cellular hypertrophy and possible synthesis of new collagen fibers may also contribute as well [93].

LA reservoir strain, as a surrogate of LA compliance, is a predictor of AF-related outcomes, including stroke [94,95]. In a study with 1361 patients, impaired LA function, as measured by reduced LA reservoir strain and higher echocardiography-derived total atrial conduction time (PA-TDI duration) at the time of AF diagnosis, was shown to be related to stroke in addition to CHA_2_DS_2_-VASc scoring [96]. Both LA volume and function in “lone AF” are predictors of cardiovascular events, including cerebral infarction or haemorrhage, hospitalization, and death [97,98]. In addition, LA deformation imaging has proved as a more robust factor of prognostication for cardiovascular outcomes, including thromboembolic events, and demonstrates the LA substrate properties more accurately than morphological parameters such as LA volume and LA ejection fraction [98,99,100]. Finally, atrial fibrosis appears to be common in IAB, and LA strain is associated with both partial and advanced IAB [101].

### 4.2. Total Atrial Conduction Time (PA-TDI Duration)

**Figure 4:**
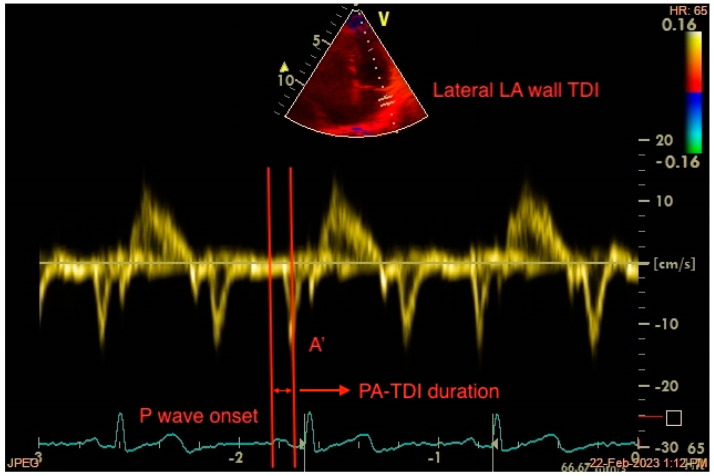
Measurement of the echocardiography-derived total atrial conduction time (PA-TDI duration) between the onset of the atrial electrical activation (P wave on ECG) and the peak of atrial mechanical contraction (A’ wave on TDI). LA: left atrium, TDI: tissue Doppler imaging.

Total atrial conduction time (PA-TDI duration) is an echocardiography-derived parameter that reflects LA electrical and structural changes. By obtaining a Tissue-Doppler Imaging (TDI) tracing of the lateral LA wall just below the mitral annulus level in the apical 4-chamber view, PA-TDI duration is calculated by measuring the time interval from the P-wave onset on the ECG to the peak of the A’-wave (Figure 4). This is the time needed for the atrial depolarization to occur and result in active atrial contraction, as assessed with TDI. Thus, PA-TDI duration represents a more complete measure of the extent of atrial remodelling than other indices [95]. 

The correlation between PA-TDI duration and the degree of right atrial appendage fibrosis was presented by Müller et al. in a histological validation study [102]. PA-TDI duration was also shown to be affected by factors known to play a significant role in atrial remodelling, such as age, hypertension, increased body mass index, valvular disease, the presence of diastolic dysfunction, and sleep apnoea [95]. Additionally, it was found to be inversely related to LA reservoir strain, a marker of reduced LA compliance [103]. A prolonged PA-TDI duration was associated with a larger LA volume index and a reduced LA reservoir strain [104]. It is an independent predictor of newly diagnosed AF [105], AF incidence after cardiac surgery [106], AF recurrence after electrical cardioversion [107], and catheter ablation, and it provides higher accuracy compared to LA volume [108]. In a prospective study of anticoagulant-naïve patients who were free of AF episodes following successful catheter ablation, prolonged PA-TDI duration was associated with an increased incidence of stroke and improved CHA_2_DS_2_-VASc score performance [109]. Another study demonstrated an independent association of PA-TDI duration with new-onset AF in hypertrophic cardiomyopathy patients, apart from other factors, such as LA dilatation and decreased LA reservoir strain [110]. Lastly, a good agreement between PA-TDI duration and total atrial conduction time obtained with an electrophysiological study was demonstrated by Erdem et al. in a study of healthy individuals [111]. 

## 5. Conclusions

Structural remodeling encounters alterations in atrial tissue properties, size, and cellular ultrastructure that underline the pathophysiological basis of AF. Electrical correlates of conduction disturbances include relative abnormalities in electrocardiographic or electrophysiological characteristics of vulnerability to AF. Finally, echocardiography- or MRI-derived functional correlates of conduction disturbances may further enhance AF prediction. 

## Figures and Tables

**Table 1:** P wave parameter abnormalities related to atrial fibrillation.

P Wave Parameters	Normal Values	Values Related to AF
P wave duration	<120 ms	≥120 ms
P wave axis	Between 0° and +75°	<0° or >+75°
P wave voltage	<2.5 mV in limb leads	≤0.1 mV in lead I
P wave area	<4 ms×mV	≥4 ms×mV
P wave terminal force in lead V1	≤0.04 mm×s	>0.04 mm×s
P wave dispersion	≤40 ms	>40 ms

## Data Availability

Not applicable.
